# Supplementary material for: Soluble PD-L1 generated by endogenous retroelement exaptation is a receptor antagonist
Source: eLife. 2019 Nov 15;8:e50256. doi: 10.7554/eLife.50256 (PMC6877088; doi:10.7554/eLife.50256)
Supplement: Transparent reporting form [file elife-50256-transrepform.docx]

***eLife’s* transparent reporting form**

We encourage authors to provide detailed information *within their submission* to facilitate the interpretation and replication of experiments. Authors can upload supporting documentation to indicate the use of appropriate reporting guidelines for health-related research (see [EQUATOR Network](http://www.equator-network.org/%20)), life science research (see the [BioSharing Information Resource](https://biosharing.org/)), or the [ARRIVE guidelines](http://www.plosbiology.org/article/info:doi/10.1371/journal.pbio.1000412) for reporting work involving animal research. Where applicable, authors should refer to any relevant reporting standards documents in this form.

If you have any questions, please consult our Journal Policies and/or contact us: [editorial@elifesciences.org](mailto:editorial@elifesciences.org).

**Sample-size estimation**

- You should state whether an appropriate sample size was computed when the study was being designed
- You should state the statistical method of sample size computation and any required assumptions
- If no explicit power analysis was used, you should describe how you decided what sample (replicate) size (number) to use

Please outline where this information can be found within the submission (e.g., sections or figure legends), or explain why this information doesn’t apply to your submission:

No power calculations to determine sample size in advance were performed as we relied on post-hoc statistical analysis (as described below and in Materials and Methods) to assess whether distributions were significantly different. Group/sample sizes for individual experiments or human studies were determined by previous pilot studies and experience regarding the experimental parameters measured.

**Replicates**

- You should report how often each experiment was performed
- You should include a definition of biological versus technical replication
- The data obtained should be provided and sufficient information should be provided to indicate the number of independent biological and/or technical replicates
- If you encountered any outliers, you should describe how these were handled
- Criteria for exclusion/inclusion of data should be clearly stated
- High-throughput sequence data should be uploaded before submission, with a private link for reviewers provided (these are available from both GEO and ArrayExpress)

Please outline where this information can be found within the submission (e.g., sections or figure legends), or explain why this information doesn’t apply to your submission:

All experiments were performed at least 3 times, and all values displayed represent results from individual experiments (i.e. biological, not technical replicates). For all human CD8+ T cell experiments, at least 3 individual PBMC donors were analyzed in separate experiments. Immunoblot and immunofluorescence images shown are representative of at least 3 independent experiments. No outliers were encountered and no exclusion/inclusion of data has been performed.

**Statistical reporting**

- Statistical analysis methods should be described and justified
- Raw data should be presented in figures whenever informative to do so (typically when N per group is less than 10)
- For each experiment, you should identify the statistical tests used, exact values of N, definitions of center, methods of multiple test correction, and dispersion and precision measures (e.g., mean, median, SD, SEM, confidence intervals; and, for the major substantive results, a measure of effect size (e.g., Pearson's r, Cohen's d)
- Report exact p-values wherever possible alongside the summary statistics and 95% confidence intervals. These should be reported for all key questions and not only when the p-value is less than 0.05.

Please outline where this information can be found within the submission (e.g., sections or figure legends), or explain why this information doesn’t apply to your submission:

The statistical tests used are described in the Statistical Analyses subsection of the Materials and Methods section. Parametric comparisons of normally distributed values that satisfied the variance criteria were made by unpaired Student’s *t*-tests or One Way Analysis of variance (ANOVA) tests. Data that did not pass the variance test were compared with non-parametric two-tailed Mann–Whitney Rank Sum tests or ANOVA on Ranks tests. For each data panel, statistical tests, p-values, and precision measures are shown on the figures or in the figure legends. The data are represented as mean ± standard error of the mean.

(For large datasets, or papers with a very large number of statistical tests, you may upload a single table file with tests, Ns, etc., with reference to sections in the manuscript.)

**Group allocation**

- Indicate how samples were allocated into experimental groups (in the case of clinical studies, please specify allocation to treatment method); if randomization was used, please also state if restricted randomization was applied
- Indicate if masking was used during group allocation, data collection and/or data analysis

Please outline where this information can be found within the submission (e.g., sections or figure legends), or explain why this information doesn’t apply to your submission:

C57BL/6J mice were assigned to different treatment groups at random from pools of genetically identical, age- and gender-matched litters, bred at the Francis Crick Institute animal facilities (as outlined in the Methods section).

**Additional data files (“source data”)**

- We encourage you to upload relevant additional data files, such as numerical data that are represented as a graph in a figure, or as a summary table
- Where provided, these should be in the most useful format, and they can be uploaded as “Source data” files linked to a main figure or table
- Include model definition files including the full list of parameters used
- Include code used for data analysis (e.g., R, MatLab)
- Avoid stating that data files are “available upon request”

Please indicate the figures or tables for which source data files have been provided:

Figure 1G-source data 1

Figure 1-figure supplement 3-source data 1

Figure 3B-source data 1
